# Protocol: Streamlined sub-protocols for floral-dip transformation and selection of transformants in *Arabidopsis thaliana*

**DOI:** 10.1186/1746-4811-5-3

**Published:** 2009-02-27

**Authors:** Amanda M Davis, Anthony Hall, Andrew J Millar, Chiarina Darrah, Seth J Davis

**Affiliations:** 1 Max Planck Institute, Cologne, Germany; 2 University of Liverpool, Liverpool, UK; 3 University of Edinburgh, Edinburgh, UK

## Abstract

Generating and identifying transformants is essential for many studies of gene function. In *Arabidopsis thaliana*, a revolutionary protocol termed floral dip is now the most widely used transformation method. Although robust, it involves a number of relatively time-consuming and laborious steps, including manipulating an *Agrobacterium tumefaciens *culture and aseptic procedures for the selection of plant lines harboring antibiotic-selection markers. Furthermore, where multiple transgenes are to be introduced, achieving this by sequential transformations over multiple generations adds significantly to the time required. To circumvent these bottlenecks, we have developed three streamlined sub-protocols. First, we find that *A. thaliana *can be transformed by dipping directly into an *A. tumefaciens *culture supplemented with surfactant, eliminating the need for media exchange to a buffered solution. Next, we illustrate that *A. thaliana *lines possessing a double-transformation event can be readily generated by simply by floral-dipping into a mixture of two *A. tumefaciens *cultures harboring distinct transformation vectors. Finally, we report an alternative method of transformant selection on chromatography sand that does not require surface sterilization of seeds. These sub-protocols, which can be used separately or in combination, save time and money, and reduce the possibility of contamination.

## Introduction

The generation of transgenic plants has allowed for new insights into gene function. The creation of the floral-dip protocol [1] markedly advanced the ease of creating transformants in *Arabidopsis thaliana*. Transformation can now be performed at very large scales leading to near saturating mutagenesis [2,3]. It has also allowed the systematic study of gene function through transgenic approaches. Nevertheless, there is room to improve this protocol.

There are three steps that we find to be both time-consuming and costly. These are, firstly, the step following growth of the *Agrobacterium tumefaciens *(recently reclassified as *Rhizobium radiobacter *[4]) strain in liquid culture. Here, the protocol calls for pelleting the culture and resuspending in a buffered media. This typically takes one hour after the time spent preparing the buffered media (itself a time and cost consuming process). A second time-consuming step occurs when two separate transgenic constructs are to be introduced into a single *A. thaliana *line. This is accomplished by transformation with the first construct, followed by "stacking" the second transgene, either by transforming the first transgenic line with a second construct, or by crossing two independently derived transgenic lines and obtaining the resultant F1 progeny. Both approaches are multi-generational to obtain the double transgenic plant, representing a significant time cost. Thirdly, in the selection of marker genes from transgenic seed in a population dominated by non-transformed lines, existing protocols for selection of lines typically requires surface sterilization of seed (a time-consuming process) and the plating of these seed on a sterile agar substrate (a costly process).

Clearly there is scope for improvements in steps within the floral-dip protocol. Evidence for this can be found in recently published protocols that target improvements in various stages of the transformation process [5-9]. Here, we present our simplified methods that are robustly effective in the generation and selection of transgenic *A. thaliana *plants. We provide a description of a bacterial-growth media that supports direct dipping and plant transformation after the trivial addition of surfactant, thereby eliminating the need to exchange bacteria from growth media to a buffer. Another improvement is a sub-protocol that allows for the simple introduction of two separate transgenes in one plant generation. Finally, we provide a sub-protocol that eliminates the need to select on sterile conditions with the discovery that chromatography sand is a suitable alternative to an agar substrate during the seed-selection process. Together, or separately, each of these sub-protocols offers time and cost savings to floral-dip transformation of *A. thaliana*.

## Materials and methods

### *Agrobacterium tumefaciens *(recently reclassified as *Rhizobium radiobacter*) cultures and culturing methods

*A. tumefaciens *strains ABI and GV3101 were both obtained from the Amasino group (University of Wisconsin-Madison, WI, USA). Bacterial transformation was as described [10]. The vectors respectively described were *CCR2:LUC*-HygR with the *hpt*-resistance gene conferring plant resistance to hygromycin [11], *CAB2:LUC *with the *nptII*-resistance gene conferring plant resistance to kanamycin [12], *CCR2:LUC*-Gent with the *aacCl*-resistance gene conferring plant resistance to gentamicin [13], and *GI:LUC*-Basta with the *BAR*-resistance gene conferring plant resistance to phosphinotricin [14]. To generate vectors for the identification of double transformation events, the FRB/Nluc and FKBP/CLuc elements were amplified by PCR from original plasmids [15] with Kpn*I *and Sac*I *restriction sites in the primers, and the resultant PCR products were digested with Kpn*I *and Sac*I *and the FRB/NLuc fragment was inserted into the Kpn*I *and Sac*I *sites of pPZP211 [16], and FRB/NLuc was similarly cut and ligated into the Kpn*I *and Sac*I *sites of pPZP221 [16]. For FRB/Nluc, the amplification primers were ATGGTACCATGGAGATGTGGCATGAAGG and ATTCAGAGCTCTCCATCCTTGTCAATCAAGGCG, and for FKBP/CLuc, the primers were ATGGTACCCTCGAGCCGCGGACTAGTATGTCCGGTTATGTA-AACAA and ATTCAGAGCTCTCATTCCAGTTTTAGAAGCTC.

Bacteria were generally grown in liquid culture at 28°C, ~250 rpm, in YEBS liquid media (1 g/L yeast extract, 5 g/L beef extract, 5 g/L sucrose, 5 g/L bacto-peptone, 0.5 g/L magnesium sulphate; adjusted pH 7). LB media was also used (10 g/L bacto-tryptone, 5 g/L yeast extract, 5 g/L sodium chloride; adjusted pH 7.5). Appropriate antibiotics were added to the respective cultures.

### PCR conditions

To detect the presence of FRB DNA, PCR was performed with the primers GAAGAGGCATCTCGTTTGTA and TAATAGAGGTCCCAGGCTTG. The product size was 221 bp. To detect the presence of FKBP DNA, PCR was performed with the primers GGGGCGGAGTGCAGGTGGAA and AAGACGAGAGTGGCATGTGG. The product size was 300 bp. The multiplex PCR involved the inclusion of the four above primers, with the appropriate plant DNA extracted as described [17]. PCR was as standard, and DNA presence was detected after electrophoresis on a 2% agarose gel.

### *Arabidopsis thaliana *growth

Flowering plants were grown essentially as described [1]. Selection of transgenic seeds on agar was on solid MS3 media [4.4 g/L Murashige & Skoog Basal Salt mixture (Sigma-Aldrich cat: M5524), 30 g/L sucrose, 0.5 g/L Monohydrate 2-(N-morpholino)ethanesufonic acid (MES); adjusted pH 5.7, 1.5% phyto agar (Duchefa Biochemie)]. Seed sterilization was as described [1], except that a 33% commercial bleach/Triton X-100 solution was used [33% DanKlorix (this is ~28 g/L sodium hypochlorite; Colgate-Palmolive; Hamburg) and 200 μL/L Triton X-100].

Selection on sand in a plastic-petri plate started with saturating chromatography sand with liquid MS0 media (1.1 g/L MS basal salt, 0.5 g/L MES; adjusted pH 5.7). The sands reported here were i) Silicon dioxide (SiO2); purum p.a.; acid purified; 40–200 mesh (84880 – Fluka), and ii) Quartz (SiO2); purum p.a.; powder; < 230 mesh (00653 – Sigma). Appropriate antibiotics were added to the MS0 solution before sand saturation. Dry seeds were sprinkled onto the wet sand, and after a two-day treatment at 4°C, plants were allowed to grow for two weeks.

## Protocols

### Sub-protocol 1: simplification of the bacterial dipping medium

#### Background

The original "vacuum infiltration" protocol begins with a physical introduction of bacteria to the flower. For this, an *A. tumefaciens *culture with saturated cell density is exchanged to a buffered media, and this is applied to flowering plants under a weak vacuum [18]. Clough and Bent (1998) developed a refined systematic protocol that eliminated the need for vacuum introduction of bacteria. This led to a substitution of the buffered media. These authors found that inclusion of sucrose in the buffered media was critical, but many other ingredients present in the "vacuum infiltration" media formulation could be omitted. Importantly in this floral-dip protocol, the inclusion of the surfactant Silwet L77 was found to be required if a vacuum-pressure was not used [1].

**Figure 1:**
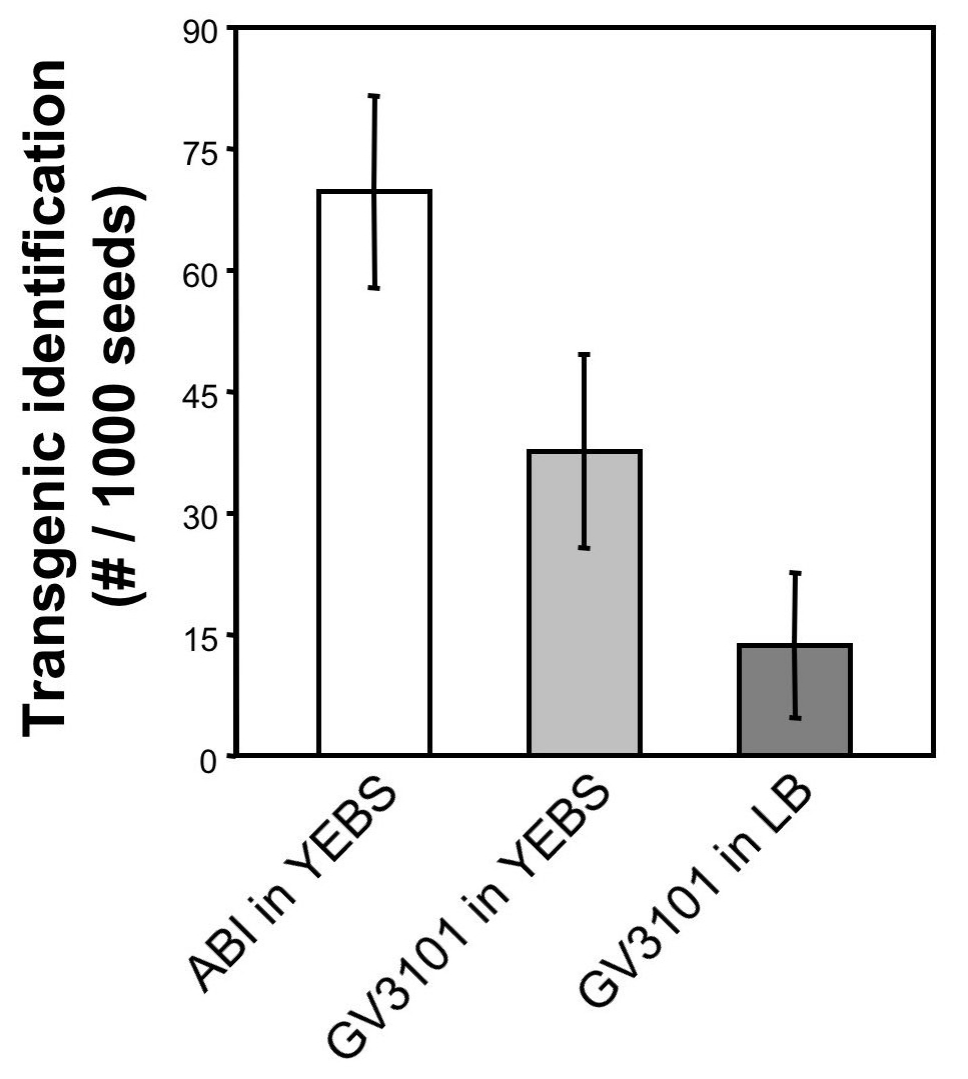
**Transformation frequencies detected after application of the 'direct dip' protocol**. *A. tumefaciens *strains ABI and GV3101 harboring the *CCR2:LUC*-HygR transgene were cultured on YEBS or LB liquid media as indicated. Floral dipping of *A. thaliana *plants was subsequently performed according to the 'direct dip' protocol, involving supplementation of the media with surfactant and sucrose. Selection of T1 seedlings was on solid MS3 medium containing hygromycin.

We sought to further simplify the dipping recipe by testing if *A. tumefaciens *grown in a standard bacterial media (YEBS) (recipe listed below) would suffice for dipping after the supplement of sucrose and Silwet L77. We found that after saturated growth in YEBS media of *A. tumefaciens *cell line ABI harboring the *CCR2:LUC *transgene [11], the addition of both 25 g per L of sucrose and 200 μL Silwet L77 per L of culture supported robust transfomation (Figure 1). Furthermore, the popular *A. tumefaciens *GV3101 cell line harboring the above-mentioned *CCR2:LUC *transgene was also robustly effective in transformation after growth in YEBS. As many labs use LB media, we tested the suitability of this media variation. Whereas the rate of transformation of *A. thaliana *transgenics was significantly lower from *A. tumefaciens *grown in LB media supplemented with 25% sucrose (w/v) and 0.02% Silwet L77, this preparation was also found to support the generation of transgenic plants (Figure 1). As another example, we transformed the above *A. tumefaciens *cell line GV3101 harboring the *CCR2::LUC *construct into a variety of *A. thaliana *accessions. Resultant transformation rates at the T1 generation were: Col-0, 1.3%; Esp-1, 0.25%; Jm-1, 0.7%; En-1, 0.6%; No-0, 0.3%; Oy-0, 0.1%; RLD, 0.9%; Tanz-1, 0.5%. Consistently and routinely, from an array of transformation constructs, and an array of *A. thaliana *genotypes and accessions, robust transformation rates between 0.1% and 10% have been observed. To date, thousands of construct/accession combinations have been dipped with the above recipe, in dozens of labs, and we have never encountered a failure in transformant identification as a result of the direct-dip protocol.

**Figure 2:**
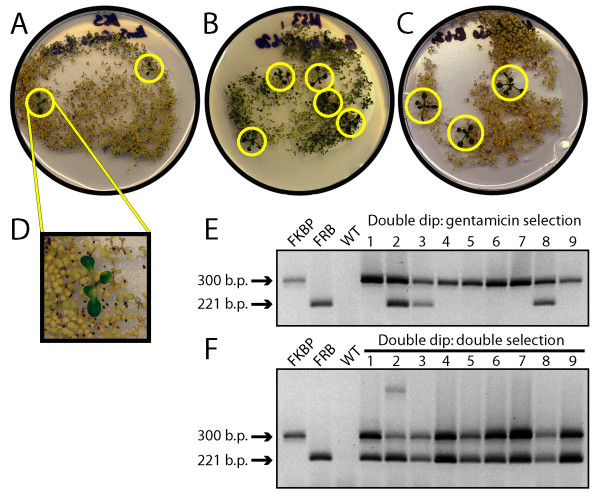
**Identification of doubly transformed *A. thaliana *lines generated using the 'double dip' protocol**. A: Growth of *A. thaliana *seedlings on MS3 plates containing both gentamicin (100 μg/mL) and kanamycin (50 μg/mL). Seeds were harvested from a mother plant that had simultaneously been transformed with respective *A. tumefaciens *ABI lines separately harboring pPZP211-FRB/NLuc (gentamicin-resistance) and pPZP221-FRB/NLuc (kanamycin-resistance). B: Growth of a replicate batch of double-dipped *A. thaliana *seedlings on gentamicin alone. C: Growth of a replicate batch of double-dipped *A. thaliana *seedlings on kanamycin alone. Circles in A-C indicate antibiotic resistant plants. D: An expanded view of a robust seedling growing on both gentamicin and kanamycin. E: Multiplex genomic PCR of FRB and FKBP sequences in genomic DNA from nine lines selected on gentamicin alone. F: Multiplex genomic PCR of FRB and FKBP sequences in nine lines selected on both antibiotics. "FKB" indicates the PCR product obtained from a known kanamycin-resistant transgenic line; "FRB" indicates the PCR product obtained from a known gentamicin-resistant transgenic line; "WT" represents the negative control using a non-transgenic line.

Motivated from the multiple media formulations that facilitated transformation, we tested whether the addition of Silwet L77 in the absence of supplemented sucrose would also be effective to generate transgenic plants after dipping, and found that simply adding the detergent to the bacteria culture, when grown in YEBS, was sufficient for floral-dip transformation (data not shown, and below protocol #2, and Figure 2). Overall, the transformation rates reported here were similar to the transformation yield of the traditional floral-dip protocol [1,6,9]. As such, we now consider the need to exchange growth media to a buffered solution to be entirely eliminated.

#### *Direct dip *protocol

This protocol presupposes that a suitable transgenic *A. tumefaciens *cell line has already been generated. If assistance is needed on how to generate a construction for plant transformation, or how to transform *A. tumefaciens *with this vector, we direct the reader to [8,10,19].

**1**. Identify transformed *A. tumefaciens *cells from an agar plate.

**2**. Start a 50 mL culture in YEBS with a bacterial streak from this transformation. Use appropriate antibiotics for bacterial selection of the Ti-plasmid and the introduced transformation vector. Grow this culture at 28–30°C until cell density is saturated (typically 2–3 days). NOTE:*Room temperature growth on a rotating lab shaker is possible if an incubator is not available*.

**3**. Pour the entire 50 mL culture into 450 mL of YEBS. Further antibiotics for bacterial selection are not required, but addition will not interfere with subsequent steps. Grow for ~8 hours NOTE:*Start the 500 mL culture in the morning and it will be ready for plant dipping just before the end of the working day*.

**4**. Add and mix 100–200 μL Silwett L77 to the culture; volume of surfactant is plant-genotype dependent. NOTE: *if a larger volume of *A. tumefaciens-*dipping solution is needed, the final saturated culture can be diluted to 1 L with distilled water supplemented with 5% (w/v) sucrose concurrent with doubling the amount of Silwet L77. No effects on transformation efficiency have been noted*.

**5**. This simple solution is ready for dipping of *A. thaliana *plants. Briefly, grow 6–10 plants per pot in 2.5"/2.5" pots, or any other suited growing container. Dip *A. thaliana *lines that have bolted, where visible flowers are present. Recent papers graphically illustrate this dipping process [7,9]. NOTE:*in slightly over-grown plants, existing siliques can be excised before dipping*.

**6**. Seal plants in closed plastic bags overnight to increase humidity, which promotes transgenic yield. Remove plants from their sealed environment before 24 hours have passed. It is critical that the enclosure is not left over 24 hours, as extended humidity in the presence of *A. tumefaciens *leads to plant death. NOTE: *as previously noted *[1]*, plants can be re-dipped every 3–5 d resulting an increase in transformation percentage, but a modest decrease in total seed yield. Our standard practice is to dip twice, with the four-day interval separating the two interventions*.

**7**. Let the plants grow until dry seeds are ready for harvesting and collect these seeds.

**8**. Aseptically select seeds for transformation, as reported [1,6,7,9], or under non-sterile conditions, as described below.

### Sub-protocol 2: simultaneous transformation of *A. thaliana *with two transformation vectors

#### Background

The insertion of more than one transgenic construct into the *A. thaliana *genome is necessary for several *in vivo *assays of protein interaction, such as BRET, FRET, and protein-fragment-complementation assays (PCA), and co-localization studies using fluorescent proteins [20-23]. Reconstruction of biochemical pathways can also require multiple tranformation events [24]. For these reasons, we sought to decrease the time to generate doubly transformed *A. thaliana *lines after the above "direct dip." We describe here a simple floral-dipping sub-protocol by which two separate transgenes can be inserted to a plant in a single *A. thaliana *generation, thus saving considerable time and resources.

Floral-dipping with a mixture of two separately grown *A. tumefaciens *cultures, each harboring a different transformation construct, can generate double transformants. Shown in Figure 2 are T1 transformants selected on media containing two selection agents, one for each construct. Note that in T1 seed from a double-dipped plant where only one transgene is selected for, double transgenics were still routinely detected (Figure 2E). When such T1 seed were subjected to double selection, only double transformants were found (Figure 2F). Not surprisingly, the rate of double transformation events is less frequent than single-transformation events, by a factor of ~4–8 (data not shown). We cannot speculate on why the double transformation rate is this high. The rate is more than sufficient to obtain an adequate number of double transformants for further analysis.

#### *Double dip *Protocol

**1**. Transform and individually select under antibiotic selection two *A. tumefaciens *lines each harboring the relevant vector. As for an equivalent "single dipping" method performed in series, each vector should confer a different resistance gene to the plant.

**2**. Prepare the two *A. tumefaciens *cultures by the method outlined above. If a final volume of 500 mL is required, add the starter 50 mL culture to 200 mL YEBS for ~8 h growth, giving a total volume of 250 mL for each individual culture. The cultures are not mixed at this stage, and are grown in separate culture flasks.

**3**. Mix the *A. tumefaciens *cultures together, resulting in 500 mL of bacterial cells in YEBS. Add 100–200 μL Silwet-77, as described above.

**4**. Proceed with floral dipping according to the method previously outlined above.

**5**. Select for double transformants on a suitable support substrate containing both of the relevant antibiotics/herbicides at appropriate selective concentrations.

### Sub-protocol 3: selecting *A. thaliana *transgenics on sand

#### Background

One limitation in the identification of transgenic *A. thaliana *lines after floral dip is that the seeds are often internally contaminated with the *A. tumefaciens *line used. Furthermore, there is often fungal contamination within the seed. For these reasons, we wished to establish a protocol for antibiotic selection that could be used under non-aseptic conditions. As an added benefit, this would eliminate the time needed to surface-sterilize seed prior to agar culture. In pilot experiments, we tried using various concentrations of antibiotics with soil-grown plants carrying different antibiotic-resistance genes. We were never successful in identifying transformants on soil. We wondered if a simpler substrate would suffice, and therefore, we attempted to select on chromatography paper *A. thaliana *transformed with the above described *CCR2:LUC *reporter line (which generates linked hygromycin resistance). This was successful (data not shown), but daily monitoring of water levels was required given the limited surface area of paper as a growth substrate. There was thus no attempt to establish a generic protocol for selection on chromatography paper. Nevertheless, we were confident that it was possible to select for an antibiotic resistance marker under non-sterile conditions.

**Figure 3:**
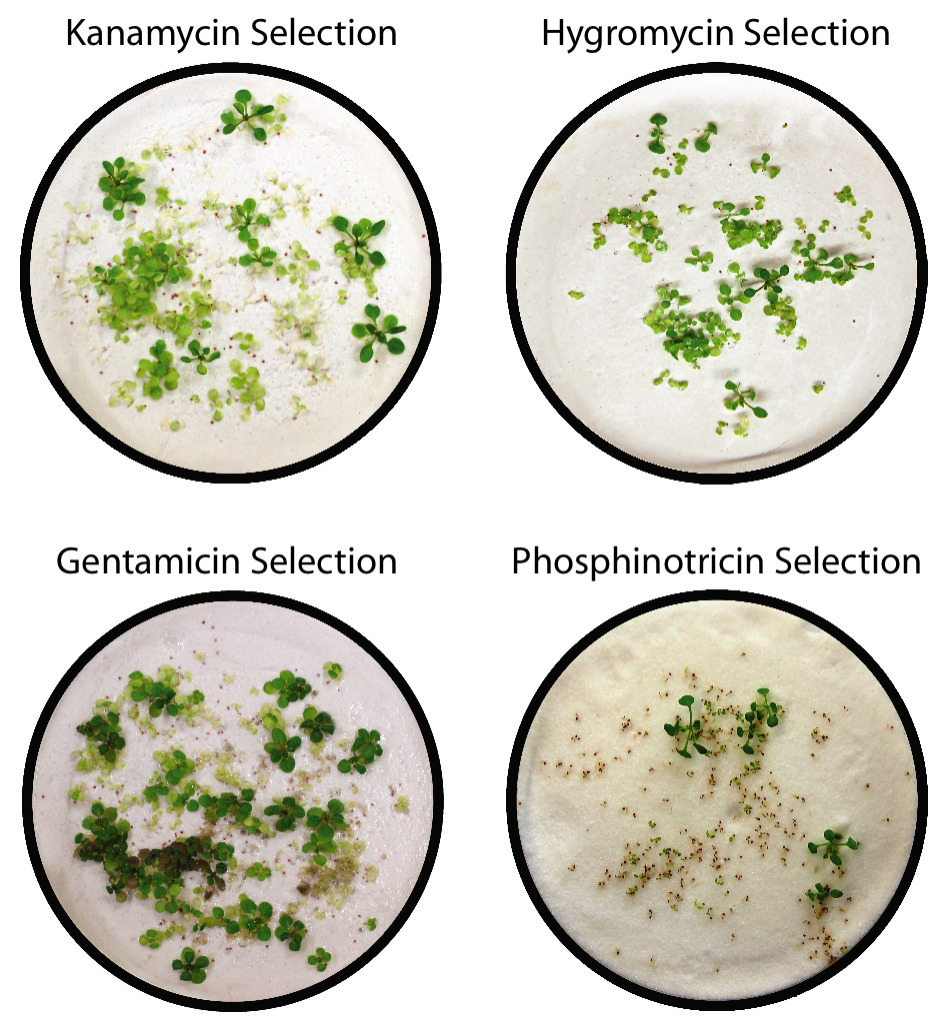
**Selection of *A. thaliana *transformants on sand**. The image shows seedlings grown on chromatography sand respectively under selection with kanamycin (100 μg/mL), hygromycin (60 μg/mL), gentamicin (125 μg/mL) and phosphinotricin (12.5 μg/mL). Note the readily identifiable transformants.

To establish a general substrate for non-aseptic selection of transgenics, we reasoned that sand might be an alternative over paper. In pilot experiments of horticultural sand, we found considerable variability to establish a working protocol. In particular, it was noticed that pre-washing the sand improved the selection ability. This led us to test pre-washed ground quartz, a chromatography sand that is readily available from a number of commercial sources. We tested a variety of grades and granulation sizes and found that dry quartz sand (Sigma catalogue no. 00653) or dry silicon dioxide (Fluka catalogue no. 84880) worked well for a wide range of antibiotic/herbicide selective agents. Shown in Figure 3 is the ability of four different marker genes to be selected on chromatography sand. The vectors used for this transformation experiment are described in the Methods section. To date, every selective agent tested was effective in transgenic identification on chromatography sand.

The described techniques for sand selection can be more easily scaled up compared to conventional transformant selection, simply by using more sand for larger screens that require more total seed. In particular, we note that chromatography sand can be purchased for a lower cost compared to agar substrates. Similarly, this selection technique can be used in classrooms that do not have access to sterile conditions. Finally, the success rate of transformant identification and resultant plant survival in the sand technique can be higher than that obtained from selection on sterile agar plates, because transformants are not readily contaminated with microorganisms.

#### *Sandy way *Protocol

**1**. Place ~20 mL dry quartz sand (Sigma #00653) or dry Silicon dioxide (Fluka #84880) in a 94 mm Petri dish. NOTE:*Alternative chromatography sands can work, but the specific type of sand matters a great deal, as some sands were found to be entirely unsuitable for certain antibiotic-selection regimes. The above two sands are our favorites, but by no means do these limit potential substrate choices. A given sand must be tested empirically within any given lab for suitability of use under the given selective agent. Generally, we found that the more 'white' the sand, the greater the number of selective agents capable of supporting transformant selection*.

**2**. Saturate the sand by pipetting ~10 mL 1/4 MS Basal Salt media (without sucrose) that is buffered and pH-adjusted containing the selecting antibiotic. NOTE:*the range of antibiotic/herbicide added to the MS solution before saturating sand can often be up to twice that usually added for standard agar selection, but this must be empirically tested within the lab*.

**3**. Evenly disperse the wet sand by gently tapping the Petri dish against the lab bench. This distributes the muddy sand mixture and releases trapped air bubbles. Then pipette or decant off excess liquid media such that the wet sand is no longer muddy.

**4**. Carefully tap up to 100μL dry seed onto the wet sand. NOTE:*too much seed can result in unwanted fungal contamination, and furthermore, identification of transformants can be a problematic when within a dense seedling canopy*.

**5**. Stratify plates at 4°C for approximately two days, depending on genotype.

**6**. Move plates to a growth cabinet, as typical for agar selection. NOTE: *surgical tape can be wrapped around the Petri dish to slow evaporation; do not use parafilm as the lack of sucrose in the sand requires that the plants are dependent on CO_2 _as a carbon source for growth*.

**7**. Approximately every 3 d, open the lid of the Petri dish and add 1 to 5 mL 1/4 MS Basal Salt media, or water, such that the plate is adequately wet. Do not over-water. NOTE:*under some selection conditions it might be necessary to add a second round of antibiotic treatment. This must be established empirically*.

**8**. After 10–14 d, transformed plants should be easily identifiable. NOTE:*failure to successfully identify transgenics means that the concentration of the selective agent needs to be modified and/or a different sand substrate needs to be tested*.

**9**. To remove selected transgenics, with their now sand-embedded roots, gently pipette ~10 mL water to the sand to mildly flood the plate. The selected transgenic seedlings can now be easily removed with forceps and transferred to soil for further growth. NOTE:*we additionally use the sand-selection method for generations after the T1 selection. As one example, we select transgenic F1 seeds on sand*.

## Competing interests

The authors declare that they have no competing interests.

## Authors' contributions

SJD conceived the experiments. AMD developed the "sandy way" sub-protocol. AH and AJM developed the "direct dip" sub-protocol. CD developed the "double dip" sub-protocol. SJD wrote the paper.

## References

[B1] Clough SJ, Bent AF (1998). Floral dip: a simplified method for Agrobacterium-mediated transformation of Arabidopsis thaliana. Plant J.

[B2] Rosso MG, Li Y, Strizhov N, Reiss B, Dekker K, Weisshaar B (2003). An Arabidopsis thaliana T-DNA mutagenized population (GABI-Kat) for flanking sequence tag-based reverse genetics. Plant Mol Biol.

[B3] Alonso JM, Stepanova AN, Leisse TJ, Kim CJ, Chen H, Shinn P, Stevenson DK, Zimmerman J, Barajas P, Cheuk R (2003). Genome-wide insertional mutagenesis of Arabidopsis thaliana. Science.

[B4] Young JM, Kuykendall LD, Martinez-Romero E, Kerr A, Sawada H (2001). A revision of Rhizobium Frank 1889, with an emended description of the genus, and the inclusion of all species of Agrobacterium Conn 1942 and Allorhizobium undicola de Lajudie et al. 1998 as new combinations: Rhizobium radiobacter, R. rhizogenes, R. rubi, R. undicola and R. vitis. Int J Syst Evol Microbiol.

[B5] Chung MH, Chen MK, Pan SM (2000). Floral spray transformation can efficiently generate Arabidopsis transgenic plants. Transgenic Res.

[B6] Harrison SJ, Mott EK, Parsley K, Aspinall S, Gray JC, Cottage A (2006). A rapid and robust method of identifying transformed Arabidopsis thaliana seedlings following floral dip transformation. Plant Methods.

[B7] Liu NY, Zhang ZF, Yang WC (2008). Isolation of embryo-specific mutants in Arabidopsis: plant transformation. Methods Mol Biol.

[B8] Logemann E, Birkenbihl RP, Ulker B, Somssich IE (2006). An improved method for preparing Agrobacterium cells that simplifies the Arabidopsis transformation protocol. Plant Methods.

[B9] Zhang X, Henriques R, Lin SS, Niu QW, Chua NH (2006). Agrobacterium-mediated transformation of Arabidopsis thaliana using the floral dip method. Nat Protoc.

[B10] Mattanovich D, Ruker F, Machado AC, Laimer M, Regner F, Steinkellner H, Himmler G, Katinger H (1989). Efficient transformation of Agrobacterium spp. by electroporation. Nucleic Acids Res.

[B11] Doyle MR, Davis SJ, Bastow RM, McWatters HG, Kozma-Bognar L, Nagy F, Millar AJ, Amasino RM (2002). The ELF4 gene controls circadian rhythms and flowering time in Arabidopsis thaliana. Nature.

[B12] Millar AJ, Short SR, Chua NH, Kay SA (1992). A novel circadian phenotype based on firefly luciferase expression in transgenic plants. Plant Cell.

[B13] Farre EM, Harmer SL, Harmon FG, Yanovsky MJ, Kay SA (2005). Overlapping and distinct roles of PRR7 and PRR9 in the Arabidopsis circadian clock. Curr Biol.

[B14] Ding Z, Millar AJ, Davis AM, Davis SJ (2007). TIME FOR COFFEE encodes a nuclear regulator in the Arabidopsis thaliana circadian clock. Plant Cell.

[B15] Luker KE, Smith MC, Luker GD, Gammon ST, Piwnica-Worms H, Piwnica-Worms D (2004). Kinetics of regulated protein-protein interactions revealed with firefly luciferase complementation imaging in cells and living animals. Proc Natl Acad Sci USA.

[B16] Hajdukiewicz P, Svab Z, Maliga P (1994). The small, versatile pPZP family of Agrobacterium binary vectors for plant transformation. Plant Mol Biol.

[B17] Michaels SD, Amasino RM (2001). High throughput isolation of DNA and RNA in 96-well format using a paint shaker. Plant Molecular Biology Reporter.

[B18] Bechtold N, Ellis J, Pelletier G (1993). In planta Agrobacterium-mediated gene transfer by infiltration of adult Arabidopsis thaliana plants. C R Acad Sci Paris Life Sci.

[B19] de la Riva GA, González-Cabrera J, Vázquez-Padrón R, Ayra-Pardo C (1998). Agrobacterium tumefaciens: a natural tool for plant transformation. Electronic Journal of Biotechnology.

[B20] Bracha-Drori K, Shichrur K, Katz A, Oliva M, Angelovici R, Yalovsky S, Ohad N (2004). Detection of protein-protein interactions in plants using bimolecular fluorescence complementation. Plant J.

[B21] Walter M, Chaban C, Schutze K, Batistic O, Weckermann K, Nake C, Blazevic D, Grefen C, Schumacher K, Oecking C (2004). Visualization of protein interactions in living plant cells using bimolecular fluorescence complementation. Plant J.

[B22] Xu J, Scheres B (2005). Dissection of Arabidopsis ADP-RIBOSYLATION FACTOR 1 function in epidermal cell polarity. Plant Cell.

[B23] Xu X, Soutto M, Xie Q, Servick S, Subramanian C, von Arnim AG, Johnson CH (2007). Imaging protein interactions with bioluminescence resonance energy transfer (BRET) in plant and mammalian cells and tissues. Proc Natl Acad Sci USA.

[B24] Burgal J, Shockey J, Lu C, Dyer J, Larson T, Graham I, Browse J (2008). Metabolic engineering of hydroxy fatty acid production in plants: RcDGAT2 drives dramatic increases in ricinoleate levels in seed oil. Plant Biotechnol J.

